# Understanding the attitude of others by hearing action sounds: the role of the insula

**DOI:** 10.1038/s41598-019-50609-y

**Published:** 2019-10-08

**Authors:** G. Di Cesare, M. Marchi, C. Pinardi, G. Rizzolatti

**Affiliations:** 1 Istituto Italiano di Tecnologia (IIT), Department of Robotics, Brain and Cognitive Sciences (RBCS), Genova, Italy; 2 Department of Computer Science, University of Milan, Milan, Italy; 3 Consiglio nazionale delle Ricerche, Istituto di Neuroscienze, Parma, Italy; 4 Department of Medicine and Surgery, Neuroscience Unit, University of Parma, Parma, Italy

**Keywords:** Perception, Perception, Social behaviour, Social behaviour

## Abstract

During social interactions, actions and words can be expressed in different ways, for example gently, vigorously or rudely communicating the positive or negative attitude of the agent. These forms of communication are called vitality forms and play a crucial role in social relations. While the neural bases of speech and actions vitality forms have been investigated, there is no information on how we recognize others’ mood/attitude by hearing the sound of their actions. In the present fMRI study we investigated the neural basis of vitality forms while participants heard action sounds in two different conditions: sounds resulting from gentle and rude actions, sounds communicating the same actions without vitality forms (control stimuli). Results showed that hearing action sounds conveying rude and gentle vitality forms respect to the control stimuli produced a specific activation of the dorso-central insula. In addition, hearing both vitality forms action sounds and control stimuli produced the activation of the parieto-frontal circuit typically involved in the observation and the execution of arm actions. In conclusion, our data indicate that, the dorso-central insula is a key region involved in the processing of vitality forms regardless of the modality by which they are conveyed.

## Introduction

The observation of actions performed by others typically enables the observer to understand the goal of the action (what), the agent’s motor intention (why), and his/her attitude towards the observer (how). The aspect of attitude is fundamental for social interactions. For example, it allows one to appreciate whether the action is performed in a *gentle* or *rude* way, thus communicating the positive or negative attitude of the agent towards the observer.

Stern investigated these aspects of social communications from a psychological point of view and termed them “vitality affects^1^” and subsequently “vitality forms^2^”. These “forms” of communication have been examined by other authors, among them Trevarthen^3^ and Hobson and Lee^4^. In spite of the fundamental role of vitality forms in interpersonal relations, few studies have investigated their neural substrates. Our group began to address this issue a few years agò^5–8^.

In an initial fMRI study^6^, participants were asked to pay attention either to the action goal (what) or to the action vitality form (how). The results showed that the contrast between the *how* task and the *what* task revealed a specific activation of the dorso-central insula. A subsequent experiment investigated whether the activation of the dorso-central insula was present not only during vitality-form observation but also during vitality form imagination and execution^6^. The results showed that in all three conditions (observation, imagination, and execution), there was an activation of the *dorso-central* sector of the insula. Interestingly, this study demonstrated that this insula sector is endowed with a mirror mechanism^6^ similar to that described in the parieto-frontal circuit involved in the arm/hand movement control^9^.

More recently, Di Cesare and colleagues demonstrated that the dorso-central insula is activated not only when participants *observed* or *imagined* the performance of arm/hand action vitality forms but also when they *listened to action verbs* or *imagined* pronouncing them with gentle or rude vitality forms^8^. These findings clearly indicate that the dorso-central insula plays a crucial role in the processing of vitality forms regardless of the modality by which they are conveyed.

In the present fMRI study, we investigated whether, in addition to action and speech vitality forms, also action sounds conveying vitality forms *automatically* activate the insula and, in particular, its central sector. In order to assess this point, participants were instructed to pay attention to the action type regardless to the vitality forms. This instruction was used to corroborate, if the insula will be activated, the hypothesis of an automatic recognition of vitality forms. This issue is important because the auditory modality plays a fundamental role in conveying the attitudes of agents when they perform a given action. For example, upon hearing a vigorous knocking at the door, one will probably be frightened and prepare to act appropriately. In contrast, upon hearing a gentle knocking, one will probably assume a kind attitude before opening the door. As stimuli, our experiment used different action sounds resulting from actions performed in a gentle or rude way. As a control, we presented the same stimuli while masking the vitality forms.

The results showed that when participants listened to the action sounds, they exhibited an activation of the parieto-frontal circuit involved in the generation of the arm/hand actions responsible for producing those sounds. Most importantly, these data demonstrated that hearing action sounds conveying different vitality forms determined the activation of the dorso-central sector of the insula. Another interesting finding was the unexpected activation of the middle cingulate cortex upon hearing the vitality forms of action sounds. The significance of these activations is discussed below.

## Results

### Behavioral study

**Figure 1:**
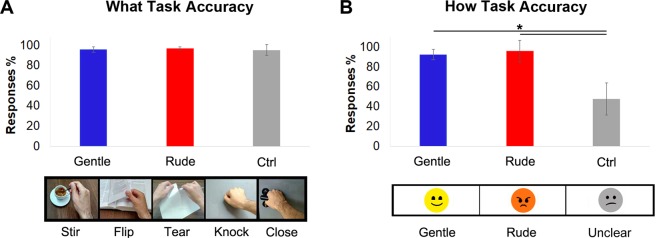
Graphs show the participants’ score obtained during the *what* (**A**) and *how* (**B**) tasks. Asterisk (*) indicates the significant comparison between conditions (*gentle, rude, control*) revealed by the post hoc analysis (p < 0.05 Bonferroni corrected). The bars indicate the standard deviation (SD). Under graphs are shown the bar used to collect the participants’ responses in the *what* and *how* tasks respectively.

A preliminary behavioral study, conducted before the fMRI experiment, was carried out in order to ascertain whether control stimuli allow participants to recognize the action type but do not the vitality form. Twenty-four healthy right-handed participants (eleven females and thirteen males, mean age = 23.7 years, SD = 2.04 years) took part in the behavioral study. Participants were presented with audio stimuli (see methods), consisting of five different action sounds: stirring the coffee, flipping through a book of paper, tearing a sheet, knocking on the door, closing the door (Fig. 1A). All the actions were presented with two vitality forms: rude and gentle (*vitality forms* condition) intermixed with control stimuli. The control stimuli consisted of the same action-sound but with the masked vitality form (for details see methods and supplementary material). Participants were required either to pay attention to the action type (*what* task) and indicate the type of action on a bar showing the five actions presented subsequently on the screen, or to pay attention to the vitality form (*how* task) of the presented action and to indicate the perceived vitality form on a second bar where three emoticons showed “*rude*,” “*gentle*,” or “*unclear*” expressions.

The participants’ responses obtained during *what* and *how* tasks were automatically computed by E-Prime software which, for each stimulus, compared the participant’s response with the correct one and assigned values “1” or “0” in case of correct or incorrect matching. Then for each participant was calculated the percentage of correct responses (hits/total responses). The correct responses obtained in the *what* and *how* tasks were then modeled using two General Linear Model (GLM). The first GLM comprised the participants’ scores related to the *what* task, for each action type presented in the *gentle*, *rude*, and *control* conditions. The second GLM comprised the participants’ scores obtained in the *how* task in the *gentle*, *rude*, and *control* conditions.

The results of the GLM analysis concerning the *what* task did not indicate any difference in stimulus recognition regardless of whether the actions were presented with or without vitality form (F = 0.37, *p* = 0.68, partial-η^2^ = 0.01; δ = 0.1). More specifically, the accuracy for the *what* task was 96% for the *gentle*, 97.2% for the *rude*, and 95.4% for the *control* condition (Fig. 1A).

The results of the GLM analysis concerning the *how* task indicated a significant difference in vitality forms recognition among the three conditions (*rude*, *gentle*, and *control*; F = 30.6, *p* < 0.001, partial-η^2^ = 0.58, δ = 1). Post hoc analysis revealed a significant difference between the *vitality forms* and *control* conditions (*gentle* > *control*, *p* < 0.001; *rude* > *control*, *p* < 0.001; Bonferroni correction; Fig. 1B). It is important to note that when participants heard the control stimuli, they judged them as *unclear* (47.6%) or randomly dubbed them as *gentle* (25.1%) or *rude* (27.3%) showing that the control stimuli did not convey vitality form information.

### fMRI study

#### Main effect of vitality and control action sounds

Fifteen healthy right-handed volunteers (seven females and eight males, mean age = 23.3, SD = 1.91) participated in the fMRI study. The stimuli presented in the fMRI study were the same as those used in the behavioral study (5 action sounds × 3 conditions [*rude*, *gentle*, *control*]). Each audio stimulus lasted 3 s. During the presentation of the stimuli, participants were asked to visually fixate on a white cross presented on a black screen and listen to the audio stimuli, paying attention to the action sounds.

**Figure 2:**
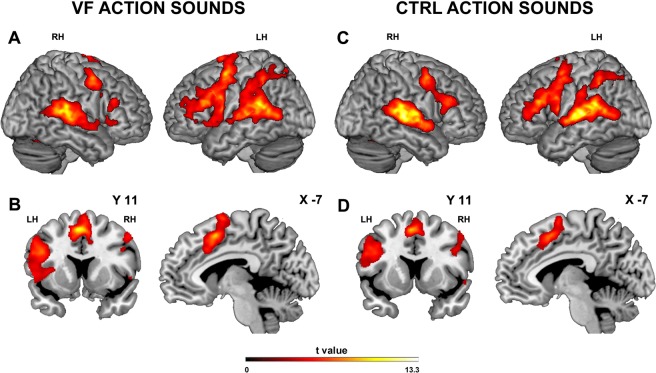
Brain activations resulting from the *vitality forms* (*gentl*e and *rude*) (**A**) and *control* (**C**) conditions vs. baseline. Coronal and parasagittal sections showing the activation of the insula and the cingulate cortex obtained in the *vitality* (**B**) and *control* (**D**) conditions vs. baseline. These activations are rendered into a standard Montreal Neurological Institute brain template (P_FWE_ < 0.05 cluster level). LH, left hemisphere; RH, right hemisphere.

Hearing action sounds conveying both gentle and rude vitality forms vs. baseline produced enhanced activation in the superior temporal gyrus bilaterally, the left inferior parietal lobule, and the premotor cortex bilaterally, with a large prevalence in the left hemisphere and left prefrontal cortex. In addition, there was a bilateral activation of the insula and of the middle cingulate cortex (aMCC) extending into the medial frontal cortex. The conjunction analysis of activations produced by gentle and rude vitality forms versus baseline is shown in Fig. 2A,B.

Hearing action sounds without vitality forms (control stimuli) determined a very similar activation pattern except for the insula cortex (Fig. 2C,D; for coordinates see Table 1). This was the major difference between stimuli with and without vitality forms.

**Table 1:** Cerebral activity during (**A**) *vitality form* listening *vs*. baseline (conjunction analysis of *gentle* and *rude*); (**B**) *control* listening *vs*.

Anatomical region	Left Hemisphere				Right Hemisphere			
	x	y	z	Z-score	x	y	z	Z-score
**(A) Vitality vs. Baseline**								
Superior temporal gyrus	−52	−38	18	7.76	62	−34	12	7.26
Posterior−Medial frontal gyrus	−7	14	45	6.92	4	6	67	5.62
Precentral gyrus	−41	0	55	6.92	51	3	51	6.23
IFG (*pars triangularis*)	−47	23	21	6.52				
Rolandic Operculum	−50	4	14	6.30				
IFG *(pars opercularis)*	−44	11	27	5.98				
Middle temporal gyrus	−54	−61	6	5.97				
Inferior parietal lobule	−42	−44	48	5.81				
Middle frontal gyrus	−27	10	49	5.60				
Middle Cingulate Cortex	−7	20	37	4.72				
Precuneus	−10	−71	41	4.28				
Cerebellum					29	−64	−26	5.21
Insula	−38	8	4	3.90				
**(B) Ctrl vs. Baseline**								
Superior temporal gyrus	−46	−41	18	Inf	65	−25	6	Inf
Heschls Gyrus	−36	−30	15	Inf				
IFG (*pars triangularis*)	−46	24	22	6.77	52	27	20	5.45
Posterior Medial Frontal Gyrus	−4	11	52	6.61				
Precentral Gyrus	−42	0	45	6.49	52	3	51	6.21
Rolandic Operculum	−51	4	14	6.24				
Cerebellum					28	−64	−26	4.68
Angular Gyrus					37	−56	52	4.12

baseline. Local maxima, as shown in Fig. 2, are given in MNI standard brain coordinates, significant threshold is set at P_FWE_ < 0.05 (cluster-level).

#### Contrasts between vitality forms and control stimuli

**Figure 3:**
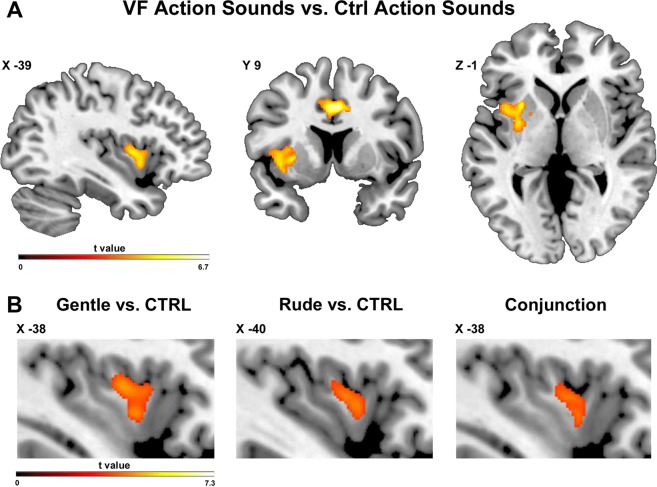
(**A**) Brain activations resulting from the main contrast vitality forms *(rude and gentle) vs. control*. (**B**) Parasagittal sections showing the left insular activations obtained in *gentle vs. control* (cluster dimension: 1671 voxels) and *rude vs. control* contrasts (cluster dimension: 859 voxels). The conjunction analysis reveals a common activation of the dorso-central sector of insula (cluster dimension: 767 voxels). These activations are rendered into a standard Montreal Neurological Institute brain template (P_FWE_ < 0.05 cluster level).

The direct contrast between vitality forms (*gentle* and *rude)* and *control* condition revealed a common activation of the left dorso-central sector of the insula and of the cingulate cortex bilaterally (Fig. 3A).

The direct contrast *gentle vs. control* revealed activation in the cingulate cortex bilaterally, the left superior parietal lobe, and most interestingly in the left dorso-central insula (Fig. 3B, left panel). The direct contrast *rude vs. control* revealed a very similar activation pattern (Fig. 3B, middle panel; for coordinates see Table 2). The conjunction analysis of the contrast *gentle vs. control* and *rude vs. control* revealed a common activation of the dorso-central insula (Fig. 3B, right panel).

**Table 2:** Cerebral activity resulting from direct contrasts: (**A**) *gentle vs. control;* (**B**) *rude vs. control*. Local maxima, as shown in Fig. 3B, are given in MNI standard brain coordinates, significant threshold is set at P_FWE_ <0.05 (cluster-level).

Anatomical region	Left Hemisphere				
	x	y	z	Z-score	Ke
**(A) Gentle vs. Ctrl**					
Middle Cingulate Cortex	−4	4	39	5.07	2070
Inferior parietal lobule	−22	−47	61	4.92	1190
Insula	−38	8	−4	4.25	1671
**(B) Rude vs. Ctrl**					
Middle Cingulate Cortex	−4	3	39	4.13	2711
Inferior parietal lobule	−22	−44	61	4.19	976
Insula	−40	9	0	4.17	859

## Discussion

The observation of actions performed by others conveys information about their goals as well as about the mood/attitude of the agent. It should be noted that in both cases, this information concerns what could be defined as the “core” understanding of the observed action (e.g. grasping, breaking ect), without any inferential implications regarding the beliefs, desires, or intentions motivating the agent’s behavior.

In the present fMRI study, we investigated whether listening to action sounds allows participants to automatically understand the mood/attitude of the agent producing that sound. In agreement with previous data of Gazzola *et al*.^10^, listening to action sound produced a bilateral activation of the parieto-frontal circuit known to be involved in action generation. It is noteworthy that, in the present study, hearing the control stimuli also produced the activation of the same circuit, as that induced by hearing the action sounds conveying vitality forms. This finding indicates that although information concerning vitality forms was absent in the control condition, participants understood the action goals.

The most interesting result of our study was that hearing gentle and rude action sounds produced the activation of the left dorso-central insula (middle and posterior short gyri). The activation of the insula, when hearing the vitality forms of action sounds, cannot be explained merely by the physical properties (loudness and duration) of the stimuli. In fact, the mean loudness of the rude stimuli was greater than that of the control stimuli, which in turn were louder than the gentle ones. Furthermore, although the duration of the control stimuli was identical to that of gentle ones, the activation of the dorso-central insula was absent when participants heard the control stimuli.

One may object that the activation of the insula was not due to the vitality forms recognition but rather to a possible effect due to a difference between natural versus unnatural stimuli. Indeed our control stimuli were characterized by the presence of a metallic sound (see supplementary material). Although we cannot completely exclude this hypothesis, previous data of Gazzola and colleagues^10^ indicate that the dorso-central sector of the insula is also activated in the contrast of hearing hand action sounds versus environmental sounds. This suggests that hand action sounds convey vitality forms regardless of the acoustical stimuli with which they are compared.

Taken together, these data suggest that the presentation of action sounds conveying vitality forms, *automatically* determines the activation of two distinct circuits. The first is represented by the parieto-frontal network, which encodes the action goal. This network is also activated by control stimuli devoid of vitality forms but conveying the action meaning. The second circuit has its central node in the dorso-central insula and is selectively activated by stimuli conveying vitality forms including action observation, speech listening, action and speech motor imagery^5–8^.

An interesting issue concernes the possible joint role of these two circuits. It is known from previous anatomical data that the dorso-central insula is connected with the parieto-frontal circuit controlling arm actions^11^. In a previous experiment we proposed that the connection between this sector of the insula and the parieto-frontal circuit may represent the neural basis for modulating the hand/arm movement according to the type of vitality form. It is plausible therefore that hearing a strong, abrupt sound elicits the preparation of the adequate motor response to the apparently unsafe context. In contrast, hearing gentle action sounds predisposes the listener to a gentle behavior.

Another interesting finding of the present study was the activation of the aMCC during the hearing of the vitality forms of action sounds which did not occur in the control condition. The functional role of the aMCC has been debated. Procyc and colleagues reported several functions for the MCC^12^, ranging from pain perception, salience, and action reward association to feedback processing and conflict monitoring^13–26^. Virtually all these data have been obtained using fMRI technique. However, it should be noted that fMRI provides only correlative data, and it may be that all these data can be explained by some more basic, underlying function. Indeed, very recent data based on intra-cortical stimulation of the aMCC (stereo-EEG) demonstrated that the stimulated patients became highly alert and exhibited behaviors characterized by the urge to act^27^. Taken together, these data appear to indicate that the aMCC activation found in the fMRI experiments during the cognitive tasks could be due to the fact that all these tasks require tension and readiness to act. The activation of aMCC in our experiment appears to be in line with this interpretation: hearing vitality forms generated by action sounds probably elicits in the listener an alarm reaction and urge to react.

## Materials and Methods

### Behavioral study

#### Subjects

A total of 24 healthy right-handed participants (eleven females and thirteen males, mean age = 23.7 years, SD = 2.04 years) took part in the behavioral study. All participants had normal or corrected-to-normal vision and normal hearing. Informed consent was obtained from all participants and the experiment was approved by the ethics committee of the University of Parma (UNIPRMR750v1) in accordance with the Declaration of Helsinki.

#### Stimuli and experimental design

**Figure 4:**
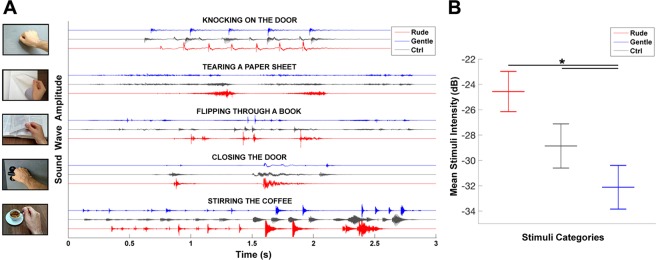
Physical characteristics of the audio stimuli. Graph A shows the sound wave amplitude for each action sound conveying (*gentle*, blue color; *rude*, red color) or not conveying (*control*, grey color) vitality forms. Graph B shows the mean intensity of audio stimuli. Asterisk (*) indicates the significant comparison between conditions (*gentle, rude, control*) revealed by the post hoc analysis (p < 0.05 Bonferroni correction).

Participants were presented with audio stimuli consisting of five different action sounds (knocking on the door, stirring the coffee, tearing a sheet of paper, flipping through a book, closing the door) performed in gentle or rude way (*vitality forms* condition). Additionally, for each action type, we presented a *control* condition consisting of the same action-sound stimuli but with the vitality form masked (Fig. 4A). The aim of the control stimuli (*control*) was to allow participants to acoustically identify the type of action without conveying any vitality form information.

All vitality form action sounds were recorded using a cardiod condenser microphone (RODE NT1), which was placed at a distance of 30 cm from the agent who performed the actions and digitized with an A/D converter module with phantom power supply (M-AUDIO M-TRACK). The audio stimuli were then processed with the software Cool Edit Pro (v2.1). It is important to note that the vitality forms of action sounds maintained their ecological loudness.

In addition, for each action type, control stimuli were constructed to allow the recognition of the action type but devoid of vitality form (for details see supplementary material). To this purpose, gentle and rude vitality forms of action sounds were overlapped and in addition, in order to be sure that some aspects of these compound stimuli provided information on vitality forms, a distortion effect was added to all control stimuli. This distortion effect was obtained by using the Cool Edit Pro software (v2.1 echo filter). Examples of the resulting control stimuli are presented in the supplementary material. Finally, the control action sounds were equated for loudness in order to match the mean perceived loudness value of the corresponding vitality forms of action sounds (for details see supplementary material). The physical characteristics of all presented audio stimuli were assessed using MATLAB. For each action sound, we reported the sound-wave amplitude and estimated the mean intensity for *rude*, *gentle*, and *control* conditions (Fig. 4A,B).

Each audio stimulus was presented in a time window of 3 s (scanner silence period). Note that, according to the action type, the stimuli had a different pattern characterized by sound burst and silent intervals (for details see Fig. 4A). A total of 15 stimuli were presented (5 action sounds × 3 conditions [*rude*, *gentle*, and *control*]). The experimental design was a 2 × 3 factorial with two levels of task (*what*, *how*) and three levels of condition (*rude*, *gentle* and *control*).

#### Paradigm and task

**Figure 5:**
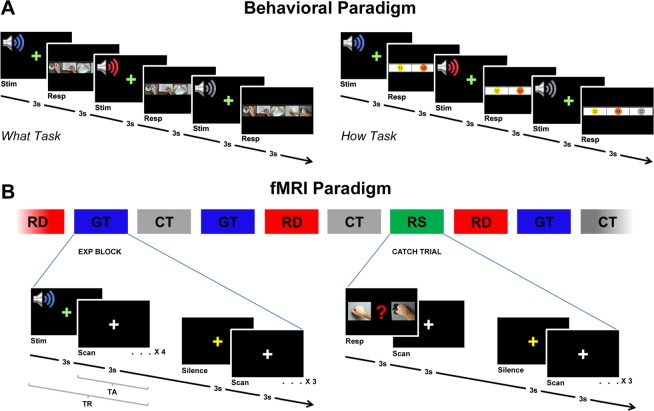
Graph shows the experimental paradigms adopted in the behavioral and fMRI experiments. (**A**) Trial timeline for what and how tasks. According with the task, after each stimulus participants were required to give an explicit response (what task: indicate the type of heard action; how task: indicate the action vitality form). (**B**) A sparse block design was used in the experiment. Audio stimuli were presented in blocks of four consecutive stimuli (duration 24 s; 4 TR) of the same condition (*gentle*, GT; *rude*, RD; *control*, CT) followed by a silent period lasting 18 s (3 TR). Intermixed with experimental blocks, in 33% of cases, were presented the catch trial blocks (RS), in which participants had to indicate the last listened action sound.

The behavioral experiment consisted of two experimental sessions. Participants were presented with gentle, rude and control action sounds and were required to pay attention to the action type (*what* task) or to the vitality form (*how* task). After the presentation of each stimulus, participants had to indicate on a bar the action type (*stir, flip, tear, knock, close*) or the perceived vitality form (*rude*, *gentle*, *unclear*) (Fig. 5A).

Using E-Prime software, a total of 15 stimuli were presented for the *what* task and for the *how* task, respectively (5 action sounds × 3 conditions [*rude*, *gentle*, *control*]). Each stimulus was presented 10 times per task. Each experimental session consisted of 150 trials presented as single events in a randomized order (Fig. 5A). The presentation order of the experimental sessions was balanced across participants. Twelve participants started with the *what* task (run 1), followed by the *how* task (run 2) and twelve participants started with the *how* task (run 1), followed by the *what* task (run 2). Each session lasted about 10 min, and the entire experiment lasted about 20 min.

### fMRI study

#### Participants

A total of 15 healthy right-handed volunteers (seven females and eight males, mean age = 23.3, SD = 1.91]) participated in the experiment. All participants had normal or corrected-to-normal vision and normal hearing. They gave written informed consent to participate in the experimental procedure, which was approved by the local ethics committee (Parma).

#### Experimental design

In the experiment, a sparse block design was used^10,28^. Thirty seven sequential slices were collected for the whole brain in 3 s (acquisition time) followed by a scanner silence period lasting 3 s (TR = 6 s). Audio stimuli were presented during this period. The experimental stimuli were presented in blocks of four consecutive stimuli of the same condition (*vitality forms* [*rude*, *gentle*] or *control*) followed by a silent period of 18 s (3 TR) (see Fig. 5). Randomly intermixed with experimental blocks were presented the catch trial blocks, in which participants had to indicate the last action they heard (knocking on the door, stirring the coffee, tearing a sheet of paper, flipping through a book, or closing the door) by pressing a button. The experiment consisted of two functional runs with a total of eight blocks (32 single trials) for each condition (*rude*, *gentle*, *control*), presented in a randomized order. Each functional run lasted about 10 min.

### Paradigm and task

Participants lay in the scanner in a dimly lit environment. The stimuli were presented using a digital audio system with 30-dB noise-attenuating headset with a 40 Hz–40 kHz frequency response (VisuaSTIM). The software E-Prime 2 Professional was used to present the stimuli and to record the participants’ answers. Before the experiment, participants already laying in the scanner, performed a training session consisting of a random presentation of the five actions type presented in each condition (5 rude, 5 gentle, 5 control). The aim of this test was to ascertain the participants’ ability to recognize the actions (100% on average).

During the presentation of the stimuli, participants were asked to visually fixate on a white cross presented on a black screen and hear the audio stimuli, paying attention to the action types. In 33% of the cases, during the inter-block phase, participants were asked to indicate, using a response box placed inside the scanner, the last action they heard (catch trial) choosing between two presented actions. The catch trials were randomly presented and lasted 3 s (Fig. 5B). The analysis of the catch trials showed that the participants’ mean response accuracy was 92.5%.

#### Stimuli

The stimuli presented in the fMRI study were the same as those used in the behavioral study. Each audio stimulus was presented in a time window of 3 s. A total of 15 stimuli were presented (5 action sounds × 3 conditions [*rude*, *gentle*, *control*]).

#### fMRI data acquisition

Imaging data were collected on a 3 Tesla Discovery MR750 GE scanner equipped with an eight-channel receiver head coil. Functional images were acquired using a gradient EPI sequence with a TR of 6000 ms, TE of 30 ms, flip angle of 90°, parallel imaging acceleration factor of 2, 205 × 205 mm^2^ field of view, voxel size of 2.5 × 2.5 × 3 mm^3^. The scanning sequence comprised 102 ascending sequential volumes composed by 37 axially slices. Additionally, a high resolution T1-weighted structural image (1 × 1 × 1 mm^3^) was acquired with a TR of 8100 ms, TE of 3.2 ms, flip angle of 12° for each participant.

#### Statistical analysis

Data analysis was performed with SPM12 (Wellcome Trust Center for Neuroimaging, London, UK). The first three volumes of each run were discarded to allow T1 equilibration effects. For each participant, functional volumes were first slice-timing corrected accordingly to sparse imaging acquisition (TA: acquisition time = 3000 ms), realigned to the mean volume and unwarped for between-scan motion correction. Subsequently, the T1-weighted image was resampled into functional image space before segmentation into gray, white and cerebrospinal fluid and normalization to the Montreal Neurological Institute (MNI) space, according to SPM12 preprocessing pipeline. Finally, spatial transformations derived from segmentation step were then applied to the realigned EPIs for normalization to MNI space with a voxel size of 1 mm × 1 mm × 1 mm. At the end of preprocessing, all functional normalized volumes were then spatially smoothed with a 6-mm full-width half maximum isotropic Gaussian kernel. For all subjects, head motion was carefully checked and no participant has met the exclusion criteria of 3 mm mean displacement. Data were analyzed using a random-effects model^29^, implemented in a two-level procedure. At the first level (single subject analysis), the BOLD signal was modeled using a general linear model (GLM) comprising the onsets and durations of each event for each functional run. The GLM model consisted of four regressors: *rude*, *gentle*, *control*, and *response*. Audio stimuli were presented in blocks of four consecutive stimuli of the same condition. Within each block, the audio stimuli were modeled as a single event lasting 3 s. The catch trial intermixed with experimental blocks were modeled as single event lasting 3 s.

In the second-level analysis (group analysis), for each participant, the contrast images of the first level were entered into a flexible factorial model. This model consisted of three regressors (*rude*, *gentle*, *control*) and considered the activation patterns resulting from the contrast between conditions (*rude vs. control*, *gentle vs. control*). In addition, to highlight voxels activated in both *gentle vs. control* and *rude vs. control* contrasts, a conjunction analysis was performed.

The location of the activation foci was determined in the stereotaxic space of the MNI coordinates system. All significant clusters were identified using an a priori voxel-wise FWE-corrected threshold of *p* < 0.05.

## Supplementary information

Supplementary Material

Stimuli
