# Supplementary material for: Understanding the attitude of others by hearing action sounds: the role of the insula
Source: Sci Rep. 2019 Oct 8;9:14430. doi: 10.1038/s41598-019-50609-y (PMC6783465; doi:10.1038/s41598-019-50609-y)
Supplement: Supplementary file 1 — Supplementary Material [file 41598_2019_50609_MOESM1_ESM.docx]

**Understanding the attitude of others by hearing action sounds: the role of the insula**

G. Di Cesare^1^, M. Marchi^2^, C. Pinardi^3^, G. Rizzolatti^3,4^*

1 Istituto Italiano di Tecnologia (IIT), Department of Robotics, Brain and Cognitive Sciences (RBCS), Genova, Italy;

2 Department of Computer Science, University of Milan, Milan, Italy;

3 Consiglio nazionale delle Ricerche, Istituto di Neuroscienze, Parma, Italy.

4 Department of Medicine and Surgery, Neuroscience Unit, University of Parma, Parma, Italy

*Correspondence to [giuseppe.dicesare@unipr.it](mailto:giuseppe.dicesare@unipr.it)

Phone: + 39-0521-903880

Fax: + 39-0521-903900

**Supplementary Material**

The audio stimuli used in the present experiment were recorded using a cardiod condenser microphone (RODE NT1), which was placed at a distance of 30 cm from the agent who performed the actions and digitized with an A/D converter module with phantom power supply (M-AUDIO M-TRACK). Audio stimuli were generated as WAV uncompressed files with a sample rate of 44100Hz, sample depth 16bits, single channel, with duration of 2s corresponding to 88200 samples. The audio stimuli were then processed with the software Cool Edit Pro (v2.1) in order to present each stimulus in a time window of 3s. It is important to note that the vitality forms of action sounds maintained their ecological loudness.

For each action sound, control stimulus was created by algorithmically summing the two corresponding rude and gentle stimuli and then applying the distortion echo filter (Cool Edit Pro (v2.1) in order to mask the vitality form. Subsequently, for each control stimulus we prepared a selection of seven control stimuli characterized by a different sound intensity, ranging from high (corresponding to the rude stimulus) and low (corresponding to the gentle stimulus) intensity. For each action type, participants were required to listen to sequences of three stimuli sets (1st stimulus, rude action sound; 2nd stimulus, one of the 7 possible intensity; 3rd stimulus, gentle action sound) and indicate whether the second stimulus was perceived as in the middle or not (possible responses: yes, not, don’t know). On the bases of this test set, the control stimuli perceived as laying in middle position between gentle and rude stimuli were selected.

*Stimulus Intensity*

For each stimulus, the intensity was evaluated with Matlab (R2014b) by using the following algorithm:

1) The original signal, composed by 88200 samples of value between -1 and 1, was reduced by cutting the silence present in the initial and the final parts. The silence present in the initial part of the audio signal was selected and removed starting from the first sample to the first sample with an absolute value > 0.01. The same procedure was used to select and remove the silence present in the final part of the audio stimulus starting from the last sample with absolute value > 0.01 to the last sample of the audio signal. This cutting procedure allowed us to obtain the final audio stimuli.

2) To calculate the mean intensity of these final stimuli, for each stimulus the sample were squared and summed, and the result was divided by the number of the sample.^1,2^

In order to evaluate the differences among the stimuli intensity, the mean intensity estimated for each action sound and expressed in dB was modelled using a General Linear Model (GLM). The GLM comprised the mean intensity of each action type presented in the *gentle*, *rude*, and *control* conditions. The results of the GLM analysis indicated a difference in stimulus intensity among the three conditions (p < 0.001, partial-η2 = 0.9, δ = 1). Post hoc analysis revealed a significant difference between the vitality forms and control conditions (gentle vs. control, p < 0.001; rude vs. control, p < 0.001; gentle vs. rude, p<0.001; Bonferroni correction).

Bibliography

1. Stevens, S. S. (1957) Stevens' power law. *On the psychophysical law.Psychol. Rev.* 64, 153–181.

2. Ellermeier, W., Faulhammer, G. (2000). Empirical evaluation of axioms fundamental to Stevens's ratio-scaling approach: I. Loudness production. *Perception & Psychophysics, 62,* 1505–1511.
